# Peritoneal tuberculosis, an underestimated diagnosis: a case report

**DOI:** 10.1099/acmi.0.000753.v3

**Published:** 2024-05-21

**Authors:** Leila Laamara, Elmostafa Benaissa, Amine Achemlal, Amal Bounakhla, Fatna Bssaibis, Yassine BenLahlou, Adil Maleb, Mariama Chadli, Mostafa Elouennass

**Affiliations:** 1 Bacteriology Department, Mohammed V Military Teaching Hospital, Rabat, Morocco; 2 Faculty of Medicine and Pharmacy, Mohammed V University, Rabat, Morocco; 3 Gastroenterology Department, Mohammed V Military Teaching Hospital, Rabat, Morocco

**Keywords:** peritoneal tuberculosis, peritonitis, Xpert MTB/RIF assay

## Abstract

Tuberculosis is an infectious disease that most often affects the lungs, caused by human-to-human transmission of *Mycobacterium tuberculosis*. Peritoneal tuberculosis is an extra-pulmonary form of the disease that usually manifests as an ascitic syndrome, with or without fever, in a context of altered general condition, often in endemic areas. The diagnosis of peritoneal tuberculosis is not always easy, as the clinical signs are often insidious and unspecific. We report a case of peritoneal tuberculosis in an 18-year-old female, who had presented for 10 days with a progressive increase in abdominal volume associated with vomiting and diarrhoea.

## Data Summary

No data were generated during this research or are required for the work to be reproduced.

## Introduction

Tuberculosis is defined as a contagious microbial disease caused by an infection with *Mycobacterium tuberculosis*, also known as Koch’s bacillus (BK) [1]. Tuberculosis is still a major public health problem worldwide. It spreads through the air when infected people cough, sneeze or spit.

In recent decades, an increase in tuberculosis rates has occurred in developing countries due to population growth and the acquired immunodeficiency syndrome (AIDS) pandemic. According to the World Health Organization (WHO), the incidence of tuberculosis was 10.6 million in 2022 and it was responsible for 1.3 million deaths in the same year [2].

Peritoneal tuberculosis is an extra-pulmonary form of the disease, usually manifesting as a febrile or non-febrile ascitic syndrome in a context of altered general condition, often in endemic areas. It ranks fourth after pulmonary, lymph node and osteoarticular tuberculosis, and is characterized by clinical and paraclinical diversity [3]. The incidence of peritoneal tuberculosis was estimated to be 50–58 % of abdominal locations [4]

In Morocco, a total of 29 327 tuberculosis cases were notified and put on treatment in 2021, as part of the National Tuberculosis Control Program (PNLAT). The distribution of tuberculosis cases according to localization shows a high proportion of extra-pulmonary tuberculosis (49 %) [5].

The diagnosis of peritoneal tuberculosis is not always easy, as the clinical signs are often insidious and unspecific. It is made based on a combination of clinical, biological and radiological evidence.

We report a case of peritoneal tuberculosis in an 18-year-old female, who had been presenting with a progressive increase in abdominal volume associated with vomiting and diarrhoea.

## Case presentation

The patient was 18 years old with no particular medical history admitted to the emergency department with acute diarrhoea.

The history of the disease began 10 days before admission with a progressive increase in abdominal volume associated with vomiting and diarrhoea. All of this evolved in a context of asthenia with an unquantified fever. The patient had no personal or family history of tuberculosis.

Clinical examination on admission revealed a conscious patient in moderately maintained general condition, haemodynamically stable, pale skin and mucous membranes, and diffuse abdominal tenderness on palpation.

Initial laboratory tests on admission showed a microcytic hypochromic anaemia of 11.2 g dl^−1^, hyperleukocytosis of 12 600 µl^−1^, predominantly composed of polynuclear neutrophils, platelet count of 493 000 µl^−1^, C-reactive protein of 222.5 mg l^−1^ and a prothrombin level of 48 %. However, liver function tests were normal (Table 1).

**Table 1.** Distribution of biological parameters

Test name	Results	Units	Biological reference interval
**Haemogram**			
Haemoglobin	11.2	g dl^−1^	12–16
Red blood cell count	4.77	×10^3^ μ^−1^	3.9–5.5
MCV	70.9	fl	82–98
MCHC	23.5	pg	27–33
MCH	33.1	g dl^−1^	32–36
Absolute leucocyte count	12.6	×10^3^ μl^−1^	4–10
Absolute neutrophil count	10.8	×10^3^ μl^−1^	1.5–7.5
Platelet count	493	×10^3^ μl^−1^	150–450
**Haemostasis parameters**			
Prothrombin rate	48	%	70–100
INR	1.68	Ratio	
Biochemical parameters of blood			
Total protein	84	g l^−1^	64–83
C-reactive protein	222.5	mg l^−1^	<5
**Biochemical parameters of ascites fluid**			
Sodium	134	mmol l^−1^	–
Potassium	4	mmol l^−1^	–
Chloride	103	mmol l^−1^	–
Total protein	64	g l^−1^	–

INR international normalized ratio MCH mean corpuscular volume MCHC mean corpuscular hemoglobin concentration MCV mean corpuscular volume

The biochemical parameters of the ascites fluid showed a total protein level of 64 g l^−1^ (Table 1)

**Fig. 1.**
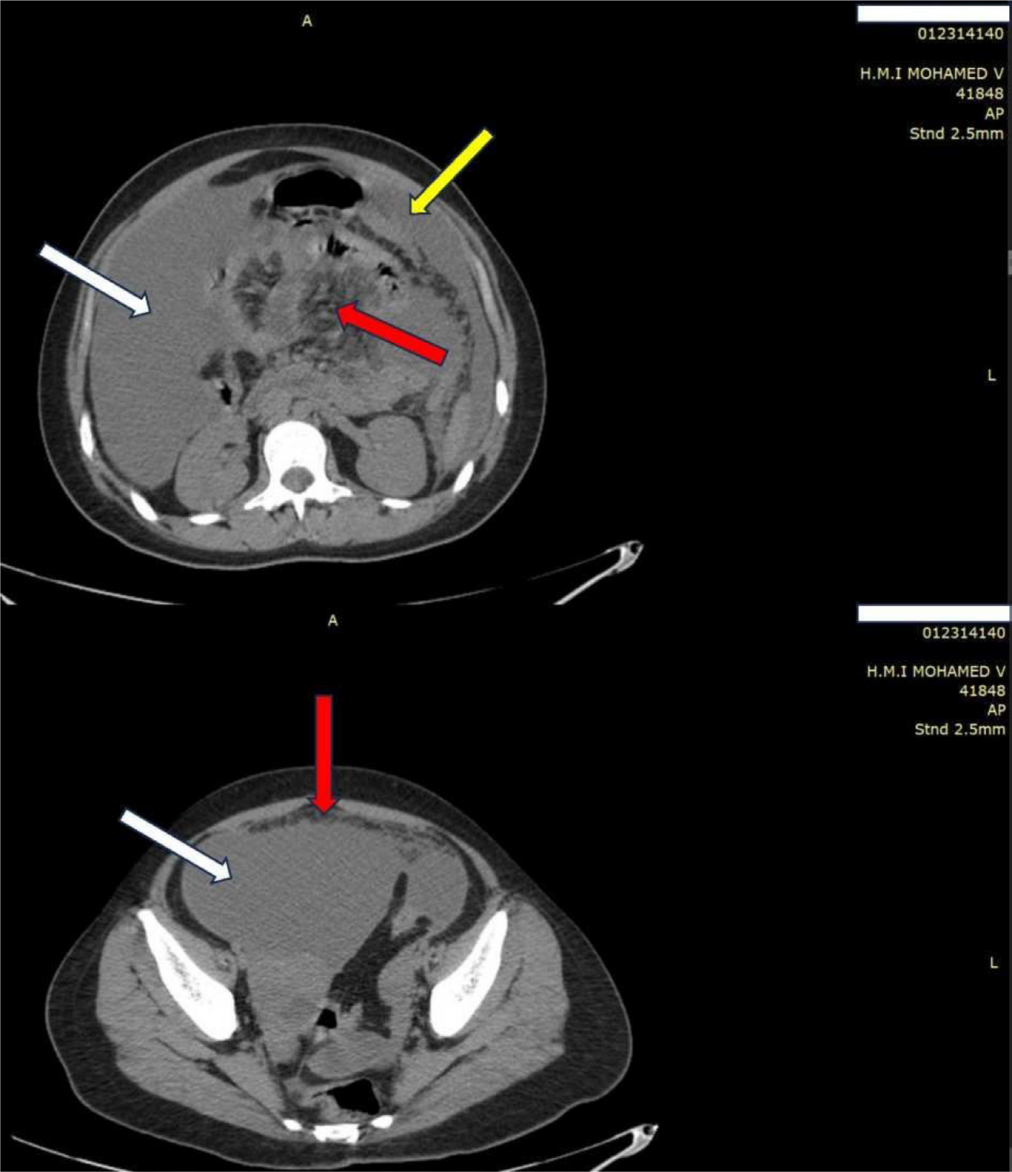
Abdominal computed tomography scan showing abundant partitioned ascites (white arrows) associated with peritoneal thickening (yellow arrow) and diffuse mesenteric infiltration (red arrows). A= Frontal view, L=Lateral view.

An abdominal computed tomography (CT) scan showed abundant partitioned ascites associated with peritoneal thickening and diffuse mesenteric infiltration (Fig. 1).

An exploratory puncture of the ascites fluid was performed, with a biochemical and bacteriological test. The ascites fluid was cloudy/turbid, yellow in colour, and had a leucocyte count of 350 mm^−3^ predominantly composed of lymphocytes (73 %) and red cell count of 1700 mm^−3^.

The samples were cultured on Columbia agar with 5 % sheep blood, on Polyvitex chocolate agar and in Brain-Heart Infusion (BHI) broth for enrichment, which was then sub-cultured onto blood agar to search for common pathogens. Incubation was performed aerobically at 37 °C for 18–24 h. After 2 days of incubation, all cultures and subcultures returned as sterile.

Due to the sterility of the cultures and the exudative nature of the ascites fluid, a real-time PCR Xpert MTB/RIF assay was conducted and detected the *Mycobacterium tuberculosis* complex at very low level with no detection of rifampicin resistance.

Mycobacterial testing was also conducted on the ascites fluid as well as a direct smear examination using the Ziehl–Neelsen staining method. Cultures on solid Löwenstein–Jensen medium and in liquid MGIT (Mycobacteria Growth Indicator Tube) medium were made. The cultures returned positive after 2 months of incubation, while direct smear examination remained negative.

As part of extensive biological assessment, a search for pulmonary tuberculosis was carried out on three consecutive pulmonary sputum samples. The culture media used for mycobacterial testing were the same as detailed above and were incubated and examined in the same manner. All of the media returned as sterile.

A diagnosis of peritoneal tuberculosis was made and the patient was put on anti-tuberculosis treatment according to Morocco’s tuberculosis protocol based on isoniazid, rifampicin, pyrazinamide and ethambutol.

The treatment was successful, resulting in significant clinical, biological and radiological improvement.

## Discussion

Peritoneal tuberculosis is the fourth most common form of extra-pulmonary tuberculosis [6]. Its incidence remains considerable in endemic areas. It represents 5–10 % of all locations and 50–58 % of abdominal locations [4]. This form affects young adults between 20 and 40 years of age [7]. A female predominance is found with abdominal locations [4].

The clinical signs of peritoneal tuberculosis are often insidious and unspecific [8], with weight loss (80 %), fever (78 %), abdominal pain (73,8 %), diarrhoea (15 %), ascites (40–100 %) and worsened general condition (76 %) [4]. Peritoneal tuberculosis should therefore be suspected in the presence of any chronic febrile abdominal pain syndrome especially in endemic areas [9].

Peritoneal tuberculosis is often caused by a rupture of a mesenteric lymph node, but can also occur through intestinal or genital contamination in immunocompromised individuals. Several contributory factors have been noted: human immunodeficiency virus, prolonged corticosteroid therapy, low socio-economic level and treatment with immunosuppressants [10].

Chest X-ray and CT scans lack specificity in extra-pulmonary tuberculosis. In fact, more than 80 % of patients do not have concomitant active tuberculosis [11]. However, even if the abdominal CT scan is not specific, it remains valuable in detecting lesions and assisting in determining their nature. In our case, the appearance of the abdominal CT scan showed abundant partitioned ascites associated with peritoneal thickening and diffuse mesenteric infiltration, raising the suspicion of a tuberculous origin.

Biological abnormalities in ascites fluid guide the diagnosis, but are not specific. An exudative, lymphocytic fluid associated with a biological inflammatory syndrome of elevated erythrocyte sedimentation rate and inflammatory proteins, as well as anaemia, is often associated with the symptomatology. In our case, laboratory tests revealed the presence of an inflammatory syndrome. Coupled with sterile cultures and the exudative nature of the ascites fluid, this prompted investigation for * M. tuberculosis* using molecular methods and as well as conventional classical methods.

It is important to note that the detection of *M. tuberculosis* complex species in ascites fluid is rarely positive through direct smear examination using the Ziehl–Neelsen staining method because samples often contain few bacteria (paucibacillary). Its sensitivity varies between 0 and 6 %. Mycobacterial culture on a specific medium such as solid Löwenstein–Jensen medium or liquid MGIT medium has a better sensitivity with a positivity rate of up to 85 % of cases, but it requires, with traditional methods, 4–8 weeks, which delays diagnosis and can worsen the prognosis [12]. In our case, the mycobacterial culture was positive after 2 months of incubation.

Several plausible biomarkers may be useful in the diagnosis of peritoneal tuberculosis, including IFN-gamma release assays, measurement of lactate dehydrogenase (LDH) activity or measurement of adenosine deaminase (ADA) activity in ascites fluid.

IFN-gamma release assays (QuantiFERON or ELISpot TB) are often useful for determination of extra pulmonary tuberculosis in immunocompetent patients. Their specificity is 97 % when the level exceeds 112 g ml^−1^ [7].

Measuring LDH in ascites fluid can make an important diagnostic contribution. It is a sensitive test for levels above 90 IU l^−1^, but its low specificity (14 %) limits its use in routine practice [12].

ADA is a ubiquitous enzyme involved in the metabolism of purine bases and is widely found in T lymphocytes, monocytes and macrophages activated during a cell-mediated inflammatory process. Measurement of ADA activity in ascites fluid has good diagnostic value when the level is ≥30 IU l^−1^ (sensitivity between 83 and 100 % and specificity between 92 and 100 %). This is a non-invasive, low-cost test that could be an alternative to invasive diagnostic tests [13]. According to a study by Saleh *et al*., mean ADA levels were significantly higher in patients with peritoneal tuberculosis than in patients with non-peritoneal tuberculosis [14]. Their study showed that a cut-off value of 35 IU l^−1^ (−1) for the ADA level produced the best results as a diagnostic test for peritoneal tuberculosis, yielding the following parameter values: sensitivity 100 %, specificity 92.6 %, positive predictive value (PPV) 87.5 % and negative predictive value (NPV) 100 %. These findings suggest that measurement of ADA activity in ascitic fluid may be used to rapidly diagnose peritoneal tuberculosis and initiate prompt treatment while waiting for a final diagnosis using the standard culture approach [14]. The unavailability of these markers limited their use in our case.

Technological progress, especially in molecular biology, has provided clinicians with new diagnostic tools for tuberculosis, including the Xpert MTB/RIF assay and GeneXpert test, approved for use since December 2010 by the WHO, which has increased sensitivity and shortened the time needed to confirm tuberculosis to just 2 h [15]. The Xpert MTB/RIF assay has also enabled the detection of rifampicin resistance, which is becoming an increasingly worrying problem, particularly in a situation where culture is not routinely available [15]. According to a prospective study conducted between October 2009 and October 2011, Clemente *et al*. analysed 1630 extra-pulmonary samples using conventional techniques (direct examination after Ziehl-Nielsen staining, followed by mycobacterial culture on solid and liquid media) and Xpert MTB/RIF assays. Of the 72 samples ultimately positive for *M. tuberculosis* (including 15 pleural fluid, 13 urine, nine lymph node biopsies and four cerebrosinal fluid), only nine (12.5 %) were positive on direct examination, while 53 (73.6 %) were positive with the Xpert MTB/RIF assay [16].

In a study conducted by Brownell *et al*., who also carried out a meta-analysis on the diagnostic performance of the Xpert MTB/RIF assay in extra-pulmonary tuberculosis, the performance of the Xpert MTB/RIF assay, compared to the result of bacterial culture as the reference technique, was 43 % for puncture fluids (pleura, ascites, cerebrosinal fluid, etc.) [17]. This molecular diagnostic tool allowed us to confirm the presence of the *M. tuberculosis* complex in this paucibacillary sample.

In peritoneal tuberculosis, differential diagnosis must be made with other causes of ascites, including inflammatory causes and also peritoneal carcinosis.

Treatment of peritoneal tuberculosis consists of two components: emergency surgical treatment of the peritonitis and anti-bacterial treatment.

## Conclusion

Peritoneal tuberculosis is still common in Morocco, and is poorly described in the literature. In an endemic context, any unusual presentation of increased abdominal volume should raise suspicions of peritoneal tuberculosis in order to ensure timely therapeutic intervention. The introduction of new tools, such as molecular biology tools, has made a major contribution to the diagnosis of pulmonary and extra-pulmonary tuberculosis. The management of peritoneal tuberculosis is multidisciplinary, requiring coordination between clinicians, bacteriologists and surgeons.

## Abbreviations

ADA: adenosine deaminase
AIDS: acquired immunodeficiency syndrome
BHI: brain-heart infusion
BK: Koch’s bacillus
CT: computed tomography
CT: computed tomography
LDH: lactate dehydrogenase
MGIT: mycobacteria growth indicator tube
MTB: *Mycobacterium tuberculosis*
WHO: World Health Organization
